# Association between self-perception of the neighborhood environment and sleep problems in older Brazilian adults: findings from ELSI-Brazil

**DOI:** 10.1590/0102-311XEN141623

**Published:** 2024-04-29

**Authors:** Daiana Vieira Sutil, Bruno de Souza Moreira, Jaquelini Betta Canever, Letícia Martins Cândido, Ana Lúcia Danielewicz, Maria Fernanda Lima-Costa, Núbia Carelli Pereira de Avelar

**Affiliations:** 1 Universidade Federal de Santa Catarina, Araranguá, Brasil.; 2 Núcleo de Estudos em Saúde Pública e Envelhecimento, Fundação Oswaldo Cruz/Universidade Federal de Minas Gerais, Belo Horizonte, Brasil.; 3 Programa de Pós-graduação em Saúde Pública, Universidade Federal de Minas Gerais, Belo Horizonte, Brasil.

**Keywords:** Aging, Aged, Sleep Quality, Neighborhood Characteristics, Envelhecimento, Idoso, Qualidade do Sono, Características da Vizinhança, Envejecimiento, Anciano, Calidad del Sueño, Características del Vecindario

## Abstract

This study aimed to investigate associations between neighborhood perception and sleep problems in older Brazilian adults. A cross-sectional study was conducted with 5,719 community-dwelling older adults (≥ 60 years) from the *Brazilian Longitudinal Study of Aging* (ELSI-Brazil, 2019-2021). The outcomes were self-reported sleep problems: poor sleep quality, daytime sleepiness, primary insomnia complaints, difficulty staying asleep, and waking up at dawn. The exposure variables were questions about the perception of participants about the physical and social environment of the neighborhood. Logistic regression was used in data analysis. Garbage, rubbish, or tall grass on the streets and the desire to move were associated with higher odds of poor sleep quality. Concern about falling due to damaged sidewalks, concern about having difficulties taking transportation, and concern about having difficulties crossing the street were associated with higher odds of all sleep problems. Sound/noise of buses and cars was associated with higher odds of some sleep problems. Perceiving the neighborhood as a good place to live was associated with lower odds of daytime sleepiness and primary insomnia complaints. Trusting most people in the neighborhood and perceiving that kids and younger people treat adults with respect were associated with lower odds of daytime sleepiness, primary insomnia complaints, and waking up at dawn. Being a good place for kids to play and raise teenagers was associated with lower odds of daytime sleepiness. These results can assist public administrators in creating urban planning policies aimed at improving neighborhood environments as a means of health promotion.

## Resumo

Este estudo teve como objetivo investigar associações entre percepções da vizinhança e problemas de sono em idosos brasileiros. Foi realizado um estudo transversal com 5.719 idosos da comunidade (≥ 60 anos) do *Estudo Longitudinal da Saúde dos Idosos Brasileiros* (ELSI-Brasil, 2019-2021). Os desfechos foram problemas de sono autorreferidos: má qualidade do sono, sonolência diurna, queixas de insônia primária, dificuldade em manter o sono e acordar na madrugada. As variáveis de exposição foram: questões sobre a percepção dos participantes sobre o ambiente físico e social da vizinhança. Regressão logística foi utilizada na análise dos dados. Lixo, sujeira ou grama alta nas ruas e o desejo de mudar de onde mora foram associados a maiores chances de má qualidade do sono. Preocupação em cair devido a calçadas em má condição, preocupação com dificuldades para usar os meios de transporte e preocupação com dificuldades para atravessar a rua foram associadas a maiores chances de todos os problemas de sono. O som/ruído dos ônibus e carros foi associado a maiores chances de alguns problemas de sono. A percepção da vizinhança como um bom lugar para morar foi associado a menores chances de sonolência diurna e queixas de insônia primária. A confiança na maioria das pessoas da vizinhança e a percepção de que crianças e jovens tratam os adultos com respeito foram associados a menores chances de sonolência diurna, queixas de insônia primária e acordar na madrugada. Um bom lugar para as crianças brincarem e para os adolescentes crescerem foi associado a menores chances de sonolência diurna. Esses resultados podem ajudar os gestores públicos na criação de políticas de planejamento urbano voltadas a melhorias nos ambientes da vizinhança como forma de promoção da saúde.

## Resumen

Este estudio tuvo como objetivo investigar las asociaciones entre las percepcion del vecindario y los problemas de sueño en ancianos brasileños. Se realizó un estudio transversal con 5.719 ancianos de la comunidad (≥ 60 años) del *Estudio Longitudinal de Salud de los Ancianos Brasileños* (ELSI-Brasil, 2019-2021). Los desenlaces fueron los siguientes: problemas de sueño autoinformados: mala calidad del sueño, somnolencia diurna, quejas de insomnio primario, dificultad para permanecer dormido y despertarse durante la madrugada. Las variables de exposición fueron las siguientes: preguntas sobre la percepción de los participantes sobre el entorno físico y social del vecindario. En el análisis de datos se utilizó la regresión logística. La basura, la suciedad o el césped alto en las calles y el deseo de cambiar el lugar donde viven se asociaron con mayores probabilidades de tener una mala calidad del sueño. La preocupación por las caídas debido a aceras en mal estado, la preocupación por las dificultades para utilizar los medios de transporte y la preocupación por las dificultades para cruzar la calle se asociaron con mayores probabilidades de sufrir todos los problemas de sueño. El ruido producido por los autobuses y automóviles se asoció con una mayor probabilidad de sufrir algunos problemas de sueño. La percepción del vecindario como un buen lugar para vivir se asoció con menores probabilidades de sufrir somnolencia diurna y quejas de insomnio primario. La confianza en la mayoría de la gente del vecindario y la percepción de que los niños y jóvenes tratan a los adultos con respeto se asociaron con menores probabilidades de sufrir somnolencia diurna, quejas de insomnio primario y despertarse durante la madrugada. Un buen lugar para que los niños jugaran y para que los adolescentes crecieran se asoció con menores probabilidades de sufrir somnolencia diurna. Estos resultados pueden ayudar a los gestores públicos a crear políticas de planificación urbana dirigidas a mejorar los entornos vecinales como forma de promover la salud.

## Introduction

The sleep duration and architecture gradually change with aging and these changes can cause alterations that directly impact the quality of life of older adults ^1^. Sleep problems have become a growing public health concern ^2^, as their prevalence is quite high, particularly in developing countries ^3^ ^,^ ^4^. In Brazil, about 15% of the adult population has sleep problems more than half of the days of the week ^4^ and the prevalence of this condition affects 36% of individuals aged 60 years and over ^5^.

Sleep problems involve disturbances in duration, initiation, efficiency, maintenance, stages, quality, and even daytime sleep behaviors ^6^. Changes in sleep patterns can contribute to several adverse health outcomes in older adults, such as dementia ^7^, depression ^8^, obesity ^9^, and cardiopulmonary diseases ^10^. Recent evidence has shown that changes in self-perception of sleep, including poor sleep quality, daytime sleepiness, primary insomnia complaints, difficulty staying asleep, or waking up at dawn, are associated with painful manifestations ^11^, disability in basic and instrumental activities of daily living, and reduced practice of physical exercises ^12^.

Several individual factors are related to sleep problems in older adults, such as sedentary behavior ^5^, physical inactivity ^4^, systemic arterial hypertension ^13^, and polypharmacy ^14^. Some studies have also observed an association between the physical and social environment characteristics of a neighborhood and sleep problems in the older population. Physical disorder (e.g., graffiti and littering in the streets) and perception of insecurity were associated with difficulty falling asleep and waking up feeling rested ^15^ ^,^ ^16^, mainly in older adults with worse socioeconomic conditions ^15^ ^,^ ^16^ ^,^ ^17^. A recent study conducted with older Japanese adults from urban and rural areas demonstrated that concern with community mobility and problems related to public services and facilities negatively influenced sleep quality ^18^. The authors observed that living in places that are difficult to walk (e.g., with hills or steps) or without shops and public places to visit was associated with worse sleep quality ^18^. However, other contextual characteristics, such as street quality, noise pollution, and difficulties taking transportation, were not evaluated. It is important to note that, in general, previous studies on this theme investigated only the sleep quality or all sleep problems together, not analyzing the different types of sleep problems separately, such as daytime sleepiness, primary insomnia complaints, difficulty staying asleep, and waking up at dawn ^16^ ^,^ ^18^.

Additionally, most studies that evaluated the association between neighborhood environment and different sleep problems in older adults come from developed countries ^15^ ^,^ ^16^ ^,^ ^18^. Therefore, the findings of these studies cannot apply to developing countries, such as Brazil, which have completely distinct contextual, socioeconomic, and technological characteristics. Therefore, the present study aimed to examine the association between self-perception of the neighborhood environment and sleep problems in a nationally representative sample of community-dwelling older Brazilian adults. Knowledge of these associations has the potential to contribute to the implementation of health education strategies and the creation of public policies to improve the neighborhood’s physical and social environment, aiming to prevent or reduce sleep problems and their consequences in this population.

## Methods

### Study design and ethical aspects

This is a cross-sectional analysis carried out with data from the second wave of the *Brazilian Longitudinal Study of Aging* (ELSI-Brazil). The ELSI-Brazil is a household-based survey conducted with a nationally representative sample of individuals aged 50 years and over. The second wave was conducted in 2019-2021 and included participants from the first wave (2015-2016) in addition to sample replacement to ensure national representativeness. To ensure that the sample represents the urban and rural areas of small, medium, and large municipalities, ELSI-Brazil adopted a multistage stratified cluster sampling, combining stratification of primary sampling units (municipalities), census tracts, and households. More details about ELSI-Brazil’s sampling and methods can be seen in previous publications ^19^ ^,^ ^20^. Other details can also be found on the survey’s homepage (http://elsi.cpqrr.fiocruz.br/). The ELSI-Brazil was approved by the Research Ethics Committee of the René Rachou Institute, Oswaldo Cruz Foundation (CAAE: 34649814.3.0000.5091), and all participants signed an informed consent form in advance of their participation in the study.

### Population and sample

The ELSI-Brazil population is composed of community-dwelling adults aged 50 and over residing in 70 municipalities across all five Brazilian macro-regions. The second wave of ELSI-Brazil included 9,949 participants. In the current study, only participants aged 60 years and over were eligible for the analyses. Thus, data from 3,020 participants were excluded due to an age criterion. In addition, we excluded data from 1,142 participants living in rural areas, as their neighborhood environment is different from those living in urban areas, and from 68 older adults whose interview was answered by a substitute informant (i.e., proxy). Therefore, 5,719 participants composed our analytical sample.

### Exposure variables

The exposure variables included 16 questions regarding the participants’ perception of the physical and social environment of the neighborhood. The concept of neighborhood was previously presented to each participant. The neighborhood was defined as the place where a person lives and performs their daily life tasks, such as going to a bakery, grocery, local shops, visiting neighbors, and going for walks. It could be also understood as the place where the participant knows most of the people.

#### Neighborhood physical environment characteristics

The questions included in this analysis were as follows: (a) physical disorder: “In your neighborhood, are there houses and buildings with graffiti on the walls, broken windows, damaged walls, or abandoned?” (yes or no), “In your neighborhood, is there garbage, rubbish, or tall grass on the streets, sidewalks, or vacant lots?” (yes or no), “In the last 3 months, have you seen rats or signs of them on the street where you live?” (yes or no); (b) concerns with community mobility: “When you leave your home, are you concerned about falling due to damaged sidewalks?” (yes or no), “When you leave your home, are you concerned about having difficulties taking the bus, subway, or train?” (yes or no), “When you leave your home, are you concerned about having difficulties crossing the street?” (yes or no); (c) noise pollution: “In your neighborhood, does the sound/noise of buses and cars bother you?” (yes or no); (d) services: “In your neighborhood, are there grocery shops, street markets, or other selling points with a variety of fresh fruits and vegetables?” (yes or no).

#### Neighborhood social environment characteristics

The questions investigated in this study were the following: (a) safety: “Thinking about crimes and violence, which of the following sentences better defines your neighborhood?” (very safe, safe, or very unsafe); (b) violence: “In the past 12 months, have you been a victim of theft/robbery or had your home broken into?” (yes or no); (c) social cohesion: “Do you believe you can trust most people in your neighborhood?” (yes, more or less, or no); (d) pleasantness: “Is your neighborhood a good place to live?” (yes, more or less, or no), “Do kids and younger people in your neighborhood treat adults with respect? (yes, more or less, or no), “Is your neighborhood a good place for kids to play and to raise teenagers?” (yes, more or less, or no), “Is it nice to walk, jog, or ride a bicycle in your neighborhood?” (yes, more or less, or no), “Would you like to move from where you live?” (yes or no).

Participants who answered “very safe” and “safe” in the question of safety dimension were grouped into the same category. Similarly, for questions of social cohesion and pleasantness dimensions, participants who answered “more or less” and “no” were grouped. The remaining questions had only two answer options, which represented different categories.

### Outcomes

The outcomes were five different types of sleep problems frequently reported by older adults and included the self-report of poor sleep quality ^21^, daytime sleepiness ^22^, primary insomnia complaints ^23^, difficulty staying asleep, and waking up at dawn ^24^.

Sleep quality was assessed using the following question: “How would you evaluate the quality of your sleep?”, with the response options: (1) very good; (2) good; (3) fair; (4) bad; (5) very bad. This variable was recategorized into poor sleep quality (response options 4 and 5) and good sleep quality (response options 1, 2, and 3).

Daytime sleepiness was assessed by the question: “How often do you wake up feeling rested in the morning?”, with the following response options: (1) most of the time; (2) sometimes; (3) never/rarely. This variable was recategorized in the presence of daytime sleepiness (response options 2 and 3) and absence of daytime sleepiness (response option 1).

Primary insomnia complaints (initial insomnia), difficulty staying asleep (intermediate insomnia), and waking up at dawn (final insomnia) were evaluated by the following questions, respectively: “How often do you have trouble falling asleep (laying down and sleeping)?”, “How often do you have trouble sleeping because you wake up during the night?”, and “How often do you have trouble sleeping because you wake up too early and cannot go back to sleep?”, with the response options: (1) most of the time; (2) sometimes; (3) never/rarely. These variables were recategorized in the presence of insomnia (response options 1 and 2) and absence of insomnia (response option 3).

### Adjustment variables

The adjustment variables considered in this study were: (1) sociodemographic characteristics: sex (male or female) ^25^ ^,^ ^26^ ^,^ ^27^, age group (60-69, 70-79, or ≥ 80 years) ^28^, education (illiterate, 1-4, 5-8, or ≥ 9 years of education) ^29^, marital status (not married or married/stable union) ^29^, time of residence in the same municipality (0-9, 10-19, 20-29, 30-39, 40-49, or ≥ 50 years), and household income per capita (< 1, ≥ 1 and < 3, or ≥ 3 Brazilian minimum wages) ^16^; (2) health characteristics: multimorbidity (yes or no) ^30^, body mass index (BMI) (underweight: < 22.0kg/m^2^, eutrophic: 22.0-27.0kg/m^2^, or overweight: > 27.0kg/m^2^) ^31^, sleeping medication (yes or no) ^32^, and cognitive function (with or without cognitive impairment) ^33^; (3) lifestyle characteristics: leisure-time physical activity level (sufficiently active or insufficiently active) ^34^.

Multimorbidity was defined as the self-reported presence of two or more previous health conditions diagnosed by a physician, including systemic arterial hypertension, diabetes mellitus, hypercholesterolemia, myocardial infarction, angina pectoris, heart failure, stroke, asthma, chronic obstructive pulmonary disease, arthritis or rheumatism, osteoporosis, chronic back problems, depression, cancer, chronic renal failure, Parkinson’s disease, and Alzheimer’s disease. Cognitive function was assessed using a battery of tests applied across countries participating in the *Health and Retirement Family Studies* ^20^. Cognitive impairment was defined when the score was < 6 points for language/executive function, < 2 points for temporal orientation, and < 3 points for combined memory (immediate and delayed memory). The physical activity level was assessed by the short version of the *International Physical Activity Questionnaire* (IPAQ). Participants who performed > 150 minutes/week of moderate physical activity or walking or > 75 minutes/week of vigorous physical activity were considered sufficiently active.

### Statistical analysis

Data analyses were performed using the statistical software Stata, version 14.0 (https://www.stata.com). In the descriptive analysis, variables were presented using relative frequency (%) and 95% confidence interval (95%CI). To investigate the association between self-perception of the neighborhood environment and sleep problems, a logistic regression analysis was performed, estimating adjusted odds ratios (OR) and 95%CI. All analyses considered the weights of the individuals and the complex sample design through the survey (*svy*) command. The significance level was set at p-value < 0.05.

## Results

Table 1 shows the sociodemographic, health, and lifestyle characteristics of the study population (5,719 participants) and according to the investigated sleep problems. Most participants were female (55%), aged between 60-69 years (55.1%), had 1-4 years of education (42.7%), were not married (55.8%), lived for ≥ 50 years in the same municipality (51.6%), had a household income per capita of < 1 minimum wage (61.1%), presented multimorbidity (60%), were overweight (52.8%), did not use sleep medication (85.4%), exhibited no cognitive impairment (67.8%), and were insufficiently active during leisure time (81.3%) (Table 1). The description of the prevalence rates of the perceived neighborhood characteristics according to sleep problems can be seen in Table 2.

**Table 1:** Description of sociodemographic, health, and lifestyle characteristics for the total sample and according to sleep problems in older adults (≥ 60 years). *Brazilian Longitudinal Study of Aging* (ELSI-Brazil), 2019-2021.

Characteristics	Total (n = 5,719) *	Poor sleep quality (n = 5,719) *	Daytime sleepiness (n = 5,719) *	Primary insomnia complaints (n = 5,717) *	Difficulty staying asleep (n = 5,711) *	Waking up at dawn (n = 5,711) *
	% (95%CI)	% (95%CI)	% (95%CI)	% (95%CI)	% (95%CI)	% (95%CI)
Sex						
Male	44.9 (42.1; 47.7)	11.2 (9.2; 13.6)	33.6 (28.7; 38.9)	41.1 (36.4; 46.0)	42.0 (37.4; 46.8)	39.7 (35.0; 44.6)
Female	55.0 (52.2; 57.8)	20.0 (17.1; 23.3)	41.4 (36.7; 46.3)	54.2 (50.5; 57.9)	53.5 (49.6; 57.3)	49.2 (45.4; 53.0)
Age group (years)						
60-69	55.1 (51.3; 58.9)	16.0 (13.7; 18.7)	38.3 (33.3; 43.5)	47.0 (42.7; 51.3)	46.8 (42.6; 50.9)	43.0 (38.7; 47.5)
70-79	30.5 (28.0; 33.2)	15.7 (12.8; 19.1)	36.9 (32.6; 41.4)	48.9 (44.3; 53.5)	49.0 (44.4; 53.6)	45.5 (40.8; 50.2)
≥ 80	14.2 (12.1; 16.5)	17.0 (12.3; 22.9)	38.6 (31.9; 45.8)	52.5 (46.1; 58.8)	53.0 (46.8; 59.1)	51.1 (45.2; 57.0)
Education (years)						
Illiterate	13.8 (10.6; 17.6)	21.1 (18.0; 24.5)	41.8 (33.9; 50.1)	53.9 (47.9; 59.7)	52.4 (46.1; 58.6)	49.0 (42.7; 55.4)
1-4	42.7 (39.2; 46.3)	17.6 (14.8; 20.8)	39.8 (34.5; 45.4)	50.7 (45.3; 56.2)	49.4 (44.9; 54.0)	47.4 (42.9; 52.0)
5-8	19.3 (17.0; 21.7)	13.0 (9.4; 17.6)	36.7 (29.2; 44.9)	45.3 (40.2; 50.4)	48.2 (42.8; 53.7)	43.8 (38.2; 49.5)
≥ 9	24.0 (20.7; 27.7)	13.4 (9.8; 18.0)	33.1 (28.1; 38.6)	43.1 (38.0; 48.3)	43.6 (38.2; 49.2)	38.5 (33.5; 43.6)
Marital status						
Not married	55.8 (52.2; 59.3)	15.0 (12.8; 17.4)	36.2 (31.5; 41.3)	46.5 (42.2; 50.9)	46.6 (42.6; 50.5)	42.7 (38.4; 47.1)
Married/Stable union	44.1 (40.6; 47.7)	17.5 (14.4; 21.0)	40.0 (34.2; 46.2)	50.6 (46.3; 54.9)	50.6 (46.2; 55.0)	47.8 (43.4; 52.2)
Time of residence in the same municipality (years)						
0-9	5.6 (4.2; 7.6)	16.2 (10.9; 23.2)	44.4 (30.7; 59.0)	49.5 (38.3; 60.7)	51.6 (38.4; 64.7)	46.5 (34.3; 59.2)
10-19	4.6 (3.6; 6.0)	18.3 (11.4; 28.0)	42.3 (34.1; 50.9)	52.0 (44.7; 59.2)	54.9 (48.2; 61.5)	46.2 (39.6; 52.8)
20-29	8.1 (5.9; 11.1)	19.4 (14.7; 25.2)	40.2 (33.0; 47.8)	46.4 (38.6; 54.4)	45.9 (38.5; 53.4)	40.1 (32.6; 48.1)
30-39	12.8 (9.8; 16.6)	15.9 (12.4; 20.2)	39.1 (30.7; 48.3)	49.6 (43.3; 55.8)	50.8 (45.2; 56.4)	46.3 (40.4; 52.4)
40-49	16.9 (14.1; 20.0)	16.3 (12.1; 21.7)	39.1 (31.5; 47.2)	47.1 (40.6; 53.7)	47.7 (40.6; 54.9)	43.4 (35.9; 51.3)
≥ 50	51.6 (45.9; 57.3)	15.3 (12.9; 18.0)	35.8 (31.6; 40.2)	48.3 (44.1; 52.5)	47.4 (42.9; 51.9)	45.5 (41.0; 50.2)
Household income per capita (minimum wages)						
< 1	61.1 (55.9; 66.2)	18.3 (15.9; 20.9)	39.5 (34.7; 44.5)	51.3 (47.4; 55.3)	49.9 (46.1; 53.6)	47.0 (43.1; 50.9)
≥ 1 and < 3	32.9 (28.7; 37.4)	14.4 (11.4; 18.0)	36.4 (30.3; 42.8)	44.8 (39.4; 50.4)	47.0 (41.9; 52.2)	43.0 (37.9; 48.3)
≥ 3	5.8 (4.2; 8.0)	8.7 (4.4; 16.4)	33.0 (19.9; 49.5)	42.9 (28.3; 58.9)	42.5 (28.4; 57.9)	36.6 (22.9; 52.9)
Multimorbidity						
Yes	60.0 (56.1; 63.9)	21.0 (18.2; 24.0)	41.8 (37.2; 46.5)	54.6 (50.2; 58.9)	56.0 (51.6; 60.3)	51.0 (46.5; 55.5)
No	39.9 (36.0; 43.8)	8.0 (6.4; 10.0)	31.5 (25.5; 38.2)	39.1 (35.0; 43.3)	36.3 (32.6; 40.2)	35.6 (31.3; 40.1)
BMI						
Underweight	11.4 (9.6; 13.4)	17.3 (13.9; 21.4)	39.0 (33.0; 45.3)	48.6 (41.9; 55.3)	46.2 (40.8; 51.7)	46.4 (41.2; 51.6)
Eutrophic	35.6 (33.5; 37.8)	15.5 (12.7; 18.9)	36.3 (31.7; 41.2)	47.2 (43.3; 51.1)	46.9 (42.6; 51.3)	44.0 (39.8; 48.2)
Overweight	52.8 (50.2; 55.4)	16.7 (14.0; 19.8)	37.1 (32.4; 41.9)	47.7 (43.9; 51.5)	48.4 (44.6; 52.3)	43.8 (40.3; 47.4)
Sleeping medication						
Yes	14.5 (12.6; 16.5)	49.7 (42.7; 56.7)	63.0 (57.2; 68.5)	84.4 (80.2; 87.9)	80.6 (76.1; 84.4)	76.7 (71.0; 81.5)
No	85.4 (83.4; 87.3)	10.4 (8.5; 12.5)	33.6 (29.3; 38.2)	42.1 (38.3; 46.1)	42.8 (39.1; 46.6)	39.5 (35.8; 43.3)
Cognitive function						
With impairment	32.1 (26.7; 38.1)	14.2 (11.3; 17.7)	38.6 (31.7; 46.0)	47.3 (41.5; 53.1)	44.8 (39.3; 50.4)	44.1 (38.5; 49.9)
Without impairment	67.8 (61.8; 73.2)	15.5 (12.7; 18.8)	34.7 (30.1; 39.7)	45.5 (41.1; 49.9)	46.3 (42.0; 50.6)	41.3 (36.7; 46.0)
Leisure-time physical activity level						
Sufficiently active	18.6 (16.1; 21.2)	14.9 (12.1; 18.3)	36.2 (29.5; 43.5)	45.6 (40.0; 51.3)	47.2 (41.4; 52.9)	44.9 (38.4; 51.5)
Insufficiently active	81.3 (78.7; 83.8)	16.3 (13.7; 19.3)	38.3 (33.9; 42.8)	48.9 (45.1; 52.8)	48.6 (44.7; 52.5)	44.9 (41.3; 48.7)

95%CI: 95% confidence interval; BMI: body mass index.

* Number of respondents, ignoring corrections according to sampling parameters.

Note: multimorbidity: the self-reported presence of two or more previous health conditions diagnosed by a physician - systemic arterial hypertension, diabetes mellitus, hypercholesterolemia, myocardial infarction, angina pectoris, heart failure, stroke, asthma, chronic obstructive pulmonary disease, arthritis or rheumatism, osteoporosis, chronic back problems, depression, cancer, chronic renal failure, Parkinson’s disease, and Alzheimer’s disease. BMI: underweight (< 22.0kg/m^2^), eutrophic (22.0-27.0kg/m^2^), and overweight (> 27.0kg/m^2^). Cognitive function: with impairment - a score of < 6 points for language/executive function, < 2 points for temporal orientation, and < 3 points for combined memory (immediate and delayed memory); without impairment - a score higher than that described in the respective domains. Leisure-time physical activity level: sufficiently active - > 150 minutes/week of moderate physical activity or walking or > 75 minutes/week of vigorous physical activity; insufficiently active - less time on the respective activities. All estimates considered the weights of the individuals and the complex sample design.

**Table 2:** Description of physical and social environment characteristics of the neighborhood according to sleep problems in older adults (≥ 60 years). *Brazilian Longitudinal Study of Aging* (ELSI-Brazil), 2019-2021.

Characteristics	Poor sleep quality (n = 5,719) *	Daytime sleepiness (n = 5,719) *	Primary insomnia complaints (n = 5,717) *	Difficulty staying asleep (n = 5,711) *	Waking up at dawn (n = 5,711) *
	% (95%CI)	% (95%CI)	% (95%CI)	% (95%CI)	% (95%CI)
Physical environment					
Physical disorder					
Houses and buildings with graffiti on the walls, broken windows, damaged walls, or abandoned	19.4 (14.0; 26.3)	36.8 (30.4; 43.7)	44.8 (37.6; 52.2)	46.8 (39.7; 54.1)	41.9 (36.1; 47.9)
Garbage, rubbish, or tall grass on the streets, sidewalks, or vacant lots	19.2 (14.9; 24.3)	40.6 (34.7; 46.9)	50.3 (44.4; 56.1)	51.1 (45.7; 56.5)	46.9 (42.1; 51.7)
Rats or signs of them on the street	19.9 (16.1; 24.4)	40.0 (34.6; 45.6)	49.0 (45.0; 53.1)	48.5 (44.5; 52.6)	44.5 (40.0; 49.1)
Concerns with community mobility					
Concern about falling due to damaged sidewalks	20.0 (16.5; 24.0)	42.1 (37.6; 46.8)	53.5 (49.2; 57.8)	54.0 (49.9; 58.0)	51.1 (47.0; 55.2)
Concern about having difficulties taking the bus, subway, or train	21.9 (18.0; 26.5)	46.5 (40.9; 52.2)	57.9 (53.7; 62.0)	57.6 (53.7; 61.5)	54.1 (50.0; 58.2)
Concern about having difficulties crossing the street	22.3 (17.8; 27.6)	45.6 (40.2; 51.0)	57.0 (52.7; 61.5)	57.8 (53.8; 61.8)	54.2 (50.4; 57.8)
Noise pollution					
Sound/Noise of buses and cars	19.7 (14.9; 25.6)	44.1 (37.0; 51.4)	54.7 (47.2; 62.0)	56.0 (48.3; 63.4)	51.2 (42.9; 59.5)
Services					
Grocery shops, street markets, or other selling points with a variety of fresh fruits and vegetables	15.7 (13.3; 18.5)	37.5 (32.6; 42.7)	48.1 (43.9; 52.3)	48.1 (44.1; 52.1)	44.6 (40.5; 48.8)
Social environment					
Safety					
Unsafe due to crime and violence	18.9 (15.2; 23.2)	41.0 (35.9; 46.2)	50.5 (42.1; 58.8)	49.9 (41.8; 58.0)	47.4 (40.5; 54.4)
Violence					
Victim of theft/robbery or had home broken into	16.0 (11.5; 21.9)	46.4 (35.8; 57.4)	50.2 (39.0; 61.4)	52.7 (42.8; 62.3)	49.1 (39.2; 59.0)
Social cohesion					
Trust most people in the neighborhood	14.8 (12.3; 17.7)	34.0 (29.7; 38.5)	45.9 (41.3; 50.6)	45.9 (41.3; 50.4)	41.9 (37.5; 46.5)
Pleasantness					
Good place to live	15.5 (13.2; 18.1)	35.6 (30.9; 40.6)	46.7 (42.5; 50.9)	46.9 (43.1; 50.8)	43.3 (39.3; 47.4)
Kids and younger people treat adults with respect	15.0 (12.5; 18.0)	34.8 (30.8; 39.1)	46.2 (42.3; 50.2)	47.0 (43.1; 51.0)	43.0 (39.1; 46.9)
Good place for kids to play and to raise teenagers	14.5 (11.8; 17.7)	34.5 (30.1; 39.2)	46.4 (42.0; 50.8)	46.0 (41.7; 50.4)	43.1 (39.1; 47.3)
Nice to walk, jog, or ride a bicycle	14.8 (12.2; 17.7)	35.6 (30.5; 40.9)	46.7 (42.2; 51.1)	46.6 (42.4; 50.8)	43.4 (39.2; 47.6)
Desire to move from where living	22.6 (17.6; 28.5)	42.4 (36.9; 48.0)	52.8 (47.6; 58.0)	52.8 (47.6; 58.0)	48.4 (42.8; 54.1)

95%CI: 95% confidence interval.

* Number of respondents, ignoring corrections according to sampling parameters.

Note: all estimates considered the weights of the individuals and the complex sample design.

**Figure 1:**
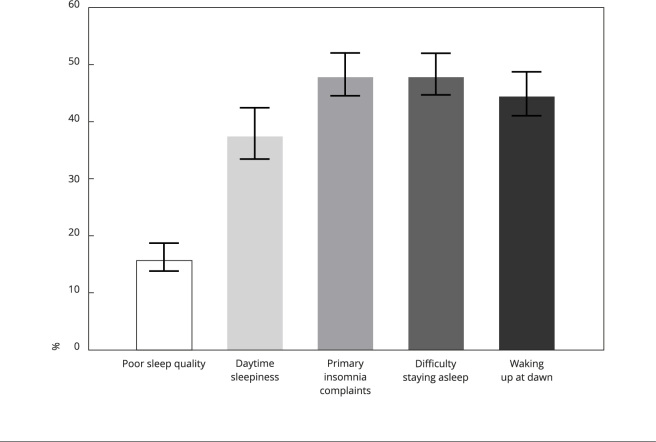
Prevalence (%) of sleep problems.

The prevalence of sleep problems was 16.1% for poor sleep quality, 37.9% for daytime sleepiness, 48.3% for primary insomnia complaints, 48.3% for difficulty staying asleep, and 44.9% for waking up at dawn (Figure 1).

The adjusted associations between the neighborhood’s physical and social environment characteristics and different types of sleep problems are described in Tables 3 and 4, respectively. According to the models, older adults who perceived their neighborhoods as having garbage, rubbish, or tall grass on the streets, sidewalks, or vacant lots presented greater odds of having poor sleep quality (OR = 1.51). Older adults who were concerned about falling due to damaged sidewalks had greater odds of presenting poor sleep quality (OR = 1.45), daytime sleepiness (OR = 1.41), primary insomnia complaints (OR = 1.41), difficulty staying asleep (OR = 1.29), and waking up at dawn (OR = 1.48). Participants who were concerned about having difficulties taking the bus, subway, or train were more likely to present poor sleep quality (OR = 1.50), daytime sleepiness (OR = 1.75), primary insomnia complaints (OR = 1.63), difficulty staying asleep (OR = 1.52), and waking up at dawn (OR = 1.66). Similarly, older adults who were concerned about having difficulties crossing the street presented higher odds of poor sleep quality (OR = 1.72), daytime sleepiness (OR = 1.58), primary insomnia complaints (OR = 1.54), difficulty staying asleep (OR = 1.51), and waking up at dawn (OR = 1.63). Moreover, participants who perceived their neighborhoods as having sound/noise of buses and cars were more likely to report poor sleep quality (OR = 1.55), difficulty staying asleep (OR = 1.51), and waking up at dawn (OR = 1.40) compared to those who did not perceive the same characteristic of neighborhood’s physical environment (Table 3).

**Table 3:** Adjusted * association between neighborhood physical environment characteristics and sleep problems in older adults (≥ 60 years). *Brazilian Longitudinal Study of Aging* (ELSI-Brazil), 2019-2021.

Characteristics	Poor sleep quality (n = 5,719) **	Daytime sleepiness (n = 5,719) **	Primary insomnia complaints (n = 5,717) **	Difficulty staying asleep (n = 5,711) **	Waking up at dawn (n = 5,711) **
	OR (95%CI)	OR (95%CI)	OR (95%CI)	OR (95%CI)	OR (95%CI)
Physical disorder					
Houses and buildings with graffiti on the walls, broken windows, damaged walls, or abandoned (vs. no)	1.55 (0.93; 2.58)	1.03 (0.72; 1.47)	0.82 (0.57; 1.18)	0.98 (0.72; 1.32)	0.93 (0.71; 1.22)
Garbage, rubbish, or tall grass on the streets, sidewalks, or vacant lots (vs. no)	1.51 (1.01; 2.25)	1.15 (0.84; 1.58)	1.15 (0.87; 1.52)	1.09 (0.86; 1.39)	1.18 (0.95; 1.48)
Rats or signs of them on the street (vs. no)	1.37 (0.99; 1.89)	1.08 (0.80; 1.45)	0.94 (0.74; 1.20)	0.88 (0.69; 1.12)	0.88 (0.70; 1.11)
Concerns with community mobility					
Concern about falling due to damaged sidewalks (vs. no)	1.45 (1.04; 2.04)	1.41 (1.08; 1.85)	1.41 (1.11; 1.77)	1.29 (1.01; 1.64)	1.48 (1.20; 1.83)
Concern about having difficulties taking the bus, subway, or train (vs. no)	1.50 (1.04; 2.15)	1.75 (1.35; 2.28)	1.63 (1.26; 2.11)	1.52 (1.22; 1.91)	1.66 (1.22; 2.25)
Concern about having difficulties crossing the street (vs. no)	1.72 (1.13; 2.61)	1.58 (1.24; 2.00)	1.54 (1.15; 2.06)	1.51 (1.19; 1.90)	1.63 (1.29; 2.06)
Noise pollution					
Sound/Noise of buses and cars (vs. no)	1.55 (1.06; 2.26)	1.31 (0.98; 1.75)	1.16 (0.90; 1.76)	1.51 (1.12; 2.02)	1.40 (1.07; 1.85)
Services					
Grocery shops, street markets, or other selling points with a variety of fresh fruits and vegetables (vs. no)	0.73 (0.46; 1.16)	0.76 (0.51; 1.12)	0.88 (0.57; 1.36)	0.90 (0.61; 1.32)	0.83 (0.55; 1.27)

95%CI: 95% confidence interval; OR: odds ratio (estimated by logistic regression).

* The model of each exposure variable was adjusted by sex, age group, education, marital status, time of residence in the same municipality, household income per capita, multimorbidity, body mass index, sleeping medication, cognitive function, and leisure-time physical activity level;

** Number of respondents, ignoring corrections according to sampling parameters.

Note: statistically significant association (p-value < 0.05). All estimates considered the weights of the individuals and the complex sample design.

**Table 4:** Adjusted * association between neighborhood social environment characteristics and sleep problems in older adults (≥ 60 years). *Brazilian Longitudinal Study of Aging* (ELSI-Brazil), 2019-2021.

Characteristics	Poor sleep quality (n = 5,719) **	Daytime sleepiness (n = 5,719) **	Primary insomnia complaints (n = 5,717) **	Difficulty staying asleep (n = 5,711) **	Waking up at dawn (n = 5,711) **
	OR (95%CI)	OR (95%CI)	OR (95%CI)	OR (95%CI)	OR (95%CI)
Safety					
Unsafe due to crime and violence (vs. safe)	1.23 (0.81 1.85)	1.23 (0.90; 1.67)	1.11 (0.75; 1.65)	0.95 (0.66; 1.37)	1.07 (0.77; 1.49)
Violence					
Victim of theft/robbery or had home broken into (vs. no)	0.71 (0.36; 1.40)	1.52 (0.89; 2.58)	1.03 (0.57; 1.84)	1.16 (0.76; 1.76)	1.06 (0.62; 1.81)
Social cohesion					
Trust most people in the neighborhood (vs. no)	0.79 (0.59; 1.05)	0.65 (0.49; 0.86)	0.71 (0.54; 0.95)	0.74 (0.54; 1.01)	0.73 (0.56; 0.96)
Pleasantness					
Good place to live (vs. no)	0.77 (0.51; 1.18)	0.57 (0.38; 0.85)	0.66 (0.44; 0.99)	0.73 (0.48; 1.09)	0.73 (0.50; 1.07)
Kids and younger people treat adults with respect (vs. no)	0.75 (0.52; 1.10)	0.64 (0.49; 0.84)	0.67 (0.50; 0.89)	0.81 (0.60; 1.09)	0.74 (0.57; 0.95)
Good place for kids to play and to raise teenagers (vs. no)	0.78 (0.55; 1.10)	0.67 (0.50; 0.90)	0.82 (0.59; 1.14)	0.79 (0.57; 1.09)	0.88 (0.63; 1.21)
Nice to walk, jog, or ride a bicycle (vs. no)	0.90 (0.61; 1.32)	0.76 (0.55; 1.06)	0.84 (0.67; 1.05)	0.87 (0.69; 1.11)	0.93 (0.74; 1.18)
Desire to move form where living (vs. no)	1.52 (1.04; 2.22)	1.10 (0.79; 1.54)	1.27 (0.99; 1.62)	1.23 (0.96; 1.58)	1.09 (0.83; 1.45)

95%CI: 95% confidence interval; OR: odds ratio (estimated by logistic regression).

* The model of each exposure variable was adjusted by sex, age group, education, marital status, time of residence in the same municipality, household income per capita, multimorbidity, body mass index, sleeping medication, cognitive function, and leisure-time physical activity level;

** Number of respondents, ignoring corrections according to sampling parameters.

Note: statistically significant association (p-value < 0.05). All estimates considered the weights of the individuals and the complex sample design.

Regarding the neighborhood social environment characteristics, older adults who reported trusting most people in their neighborhood were less likely to have daytime sleepiness (OR = 0.65), primary insomnia complaints (OR = 0.71), and waking up at dawn (OR = 0.73). Participants who perceived the neighborhood as a good place to live showed lower odds of daytime sleepiness (OR = 0.57) and primary insomnia complaints (OR = 0.66), while those who lived in neighborhoods where kids and younger people treat adults with respect were less likely to report daytime sleepiness (OR = 0.64), primary insomnia complaints (OR = 0.67), and waking up at dawn (OR = 0.74). Older adults who perceived their neighborhood as a good place for kids to play and to raise teenagers were less likely to have daytime sleepiness (OR = 0.67) and those who desired to move were more likely to present poor sleep quality (OR = 1.52) (Table 4).

## Discussion

The results from this study revealed that several characteristics of the neighborhood’s physical and social environment were associated with sleep problems among older adults living in urban areas in Brazil. After adjusting for potential confounders, environmental characteristics related to concerns with community mobility (falling due to damaged sidewalks and having difficulties taking public transportation and crossing the street) were positively associated with all the sleep problems studied. Positive associations were also observed between physical disorder (garbage, rubbish, or tall grass on the streets) and poor sleep quality, between noise pollution (sound/noise of buses and cars) and poor sleep quality, difficulty staying asleep, and waking up at dawn, and between pleasantness (desire to move from where living) and poor sleep quality. Social cohesion (trust in neighbors) was negatively associated with daytime sleepiness, primary insomnia complaints, and waking up at dawn. In addition, negative associations were observed between a good place to live and daytime sleepiness and primary insomnia complaints, between a neighborhood where kids and younger people treat adults with respect and daytime sleepiness, primary insomnia complaints, and waking up at dawn, and between a good place for kids to play and to raise teenagers and daytime sleepiness.

Older adults who reported the presence of garbage, rubbish, or tall grass on the streets, sidewalks, or vacant lots in their neighborhoods were more likely to have poor sleep quality. Unlike the present study, Watanabe et al. ^18^ did not observe an association between places with non-discarded garbage and sleep problems in older Japanese adults. This divergence across studies may be related to socioeconomic differences between the studied populations. The presence of garbage in the neighborhood in countries with great socioeconomic inequality such as Brazil could limit walking, outdoor activities and exercises, and access to public places in the neighborhood, particularly among lower-income populations ^35^. Moreover, prior research has pointed out that older adults who experience current financial hardships or financial strain throughout their lifespan have limited housing options, forcing them to reside in disordered, impoverished, densely populated, noisy, and/or unsafe neighborhoods, which can affect their sleep quality ^36^. This kind of environment, in turn, would lead to the confinement of older adults within their own homes and, consequently, would increase the time spent in sedentary behavior ^37^, a factor that is strongly associated with sleep problems, via alteration in the circadian rhythm and sleep hormone metabolism ^5^. Furthermore, confinement in one’s own home due to difficult access to streets and public places could reduce social interaction and increase stress levels, both associated with poor sleep quality ^32^.

In the present study, being concerned about falling due to damaged sidewalks was significantly associated with higher odds of all sleep problems. Corroborating our findings, Watanabe et al. ^18^ showed that the non-depressive older Japanese adults who lived in neighborhoods with few steps or hills, which make it difficult to walk in the neighborhood, had a lower prevalence of poor sleep quality (prevalence ratio - PR = 0.75; 95%CI: 0.56; 0.99). These authors investigated only sleep quality and did not explore other types of sleep problems. The presence of defects in sidewalks is well-known to be a barrier to walking and physical exercise among older adults ^38^, which can result in reduced physical activity levels and, consequently, a decreased release of nocturnal melatonin ^39^. This hormone, which is already reduced during older adults’ sleep due to the aging process, is a chronobiotic that synchronizes the individual’s intrinsic biological rhythm. Therefore, when secreted at times other than the physiological one, melatonin can lead to increased daytime sleepiness and/or insomnia ^40^, and when decreased, it can result in poor sleep quality ^41^.

Participants who were concerned about having difficulties taking the bus, subway, or train were also more likely to have all sleep problems investigated. Difficulty accessing public transport can lead to restriction in social participation and, consequently, mobility problems and greater functional dependence ^38^. Such consequences can generate sedentary behaviors and physical inactivity, which are strongly associated with sleep problems in community-dwelling older adults, as they directly influence the circadian rhythm ^5^ ^,^ ^6^.

Older adults concerned about having difficulties crossing the street were also more likely to report all sleep problems. The lack of crosswalks and reduced signaling can generate a feeling of insecurity when crossing the street due to the fear of being a victim of a traffic accident and, therefore, act as a barrier to the practice of physical activity, especially among older adults ^42^. As previously reported, the reduction in physical activity levels affects sleep via changes in hormone secretion and homeostasis of the sleep-wake cycle ^6^ ^,^ ^39^. According to Watanabe et al. ^18^, environmental changes that increase walking opportunities and provide multiple destinations can improve older adults’ sleep quality by encouraging them to leave the house regularly and enabling an active lifestyle. Thus, being able to walk in the neighborhood environment not only allows older adults to increase their activity level but also to communicate more with their neighbors, which could improve the profile of circulating cortisol throughout the day, favoring better sleep quality ^43^.

Hearing the sound/noise of buses and cars was significantly associated with poor sleep quality, difficulty staying asleep, and waking up at dawn. A previous study also found an association between living in areas with traffic noise and sleep problems in Brazilian adults (OR = 1.08; 95%CI: 1.16; 5.90) ^44^. According to the authors, sleep problems are one of the most harmful effects of noise exposure, as quality sleep is essential for daytime alertness, concentration, and good performance in daily tasks ^44^. This aspect can be even more impactful in older adults’ lives, as their sleep is usually more superficial than that of adults due to physiological changes in sleep architecture, especially in the rhythm of the sleep-wake cycle ^45^. Therefore, these noises would tend to wake up older adults more easily than adults ^46^. In addition, high noise levels can lead to malaise and indisposition ^47^ and increased blood pressure ^48^ and, consequently, cause awakening during the night, compromising older adults’ sleep quality. Other researchers claim that noise can act as an acute stressor that influences all sleep stages, especially rapid-eye-movement (REM) sleep ^49^. This effect would be more acute in older adults, as the onset of their sleep is more difficult, and the total time and efficiency of their sleep are reduced due to the aging process ^46^.

On the other hand, we observed, in general, that older adults who perceived the neighborhood environment as a good place to live, reported trusting most people in their neighborhood, and perceived that kids and younger people in their neighborhood treat adults with respect exhibited lower odds of having daytime sleepiness, primary insomnia complaints, and waking up at dawn. Corroborating our findings, previous studies have shown that living in neighborhoods with greater social cohesion (e.g., a feeling of belonging, conditions of mutual trust, shared values, and solidarity between neighbors) reduces the odds of complaints of sleep problems ^18^ ^,^ ^50^. In this sense, social cohesion acts as a protective factor because it favors the inhibition of the body’s stressor systems that negatively impact sleep through hypervigilance ^16^ ^,^ ^50^. It is also important to point out that even if sleep homeostasis is reduced with senescence, a favorable social environment perceived by older adults, in which they feel comfortable performing their daily activities, can contribute to maintaining the adequate metabolism of hormones that are essential for the sleep-wake cycle, such as growth hormone, sex hormones, thyroid stimulant, cortisol, prolactin, and melatonin ^6^.

Another finding of this study was that older adults willing to move presented higher odds of reporting poor sleep quality. Yang et al. ^51^ found that housing insecurity is strongly associated with poor sleep quality and efficiency, as this problem creates significant psychological stress and cognitive load that impact homeostatic sleep regulation, through decreased sleep deprivation during wakefulness and increased deprivation during sleep ^6^. Furthermore, the desire to move may occur due to older adults’ low sense of belonging to the place where they live, which, in turn, can lead to depressive behaviors, social isolation, feelings of loneliness, and negative emotions. This scenario can trigger concerns and psychological stress that impair the regulation of hormonal systems, which act directly on older adults’ sleep quality ^52^.

The strengths of this study include the large sample size and data from a nationally representative survey of the older Brazilian population. To our knowledge, this is the first study to analyze, at the national level, the association between self-perception of the neighborhood environment and different sleep problems in older Brazilian adults. Moreover, the present study analyzed several contextual factors, which are still little explored in studies conducted with populations from middle-high income countries, and their associations with different types of sleep problems. Despite these strengths, our findings should be interpreted with caution due to some limitations. Both sleep problems and the neighborhood’s physical and social environment characteristics were obtained subjectively; thus, the information is subject to memory, same-source, and social desirability biases. Moreover, we cannot rule out possible discrepancies between perceptions and real characteristics of the neighborhood. The models were not adjusted for race/skin color due to the strong correlation of this variable with education, which could result in overfitting. However, residual confounding is a possibility. Finally, the ELSI-Brazil’s instrument on neighborhood perception has not been validated in the Brazilian population. However, the instrument’s questions were adapted from other questions used in several studies on urban health.

## Conclusion

Several characteristics of the neighborhood’s physical and social environment were independently associated with the sleep problems analyzed. In particular, environmental characteristics related to concerns with community mobility were more consistently associated with sleep problems in community-dwelling older Brazilian adults. The current results can potentially assist to assist public managers in creating urban planning policies aimed at improving neighborhood environments. In addition, our findings can help health professionals better understand the relationship between the neighborhood environment and sleep problems, which can contribute to implementing preventive and/or management strategies for the main sleep problems reported by this population.
